# Hyperthermia‐Triggered On‐Demand Biomimetic Nanocarriers for Synergetic Photothermal and Chemotherapy

**DOI:** 10.1002/advs.201903642

**Published:** 2020-04-20

**Authors:** Junbin Gao, Fei Wang, Shuanghu Wang, Lu Liu, Kun Liu, Yicheng Ye, Zhen Wang, Hong Wang, Bin Chen, Jiamiao Jiang, Juanfeng Ou, Jan C. M. van Hest, Fei Peng, Yingfeng Tu

**Affiliations:** ^1^ School of Pharmaceutical Science Guangdong Provincial Key Laboratory of New Drug Screening Southern Medical University Guangzhou 510515 China; ^2^ School of Materials Science and Engineering Sun Yat‐Sen University Guangzhou 510275 China; ^3^ Institute for Complex Molecular Systems Eindhoven University of Technology Eindhoven 5600, MB The Netherlands

**Keywords:** cancer cell membranes, dual‐drug delivery, homotypic targeting, NIR‐triggered release, photothermal chemotherapy

## Abstract

Nanoparticle‐based drug delivery systems with low side effects and enhanced efficacy hold great potential in the treatment of various malignancies, in particular cancer; however, they are still challenging to attain. Herein, an anticancer drug delivery system based on a cisplatin (CDDP) containing nanogel, functionalized with photothermal gold nanorods (GNRs) which are electrostatically decorated with doxorubicin (DOX) is reported. The nanoparticles are formed via the crosslinking reaction of hyaluronic acid with the ancillary anticarcinogen CDDP in the presence of DOX‐decorated GNRs. The nanogel is furthermore cloaked with a cancer cell membrane, and the resulting biomimetic nanocarrier (4T1‐HANG‐GNR‐DC) shows efficient accumulation by homologous tumor targeting and possesses long‐time retention in the tumor microenvironment. Upon near‐infrared (NIR) laser irradiation, in situ photothermal therapy is conducted which further induces hyperthermia‐triggered on‐demand drug release from the nanogel reservoir to achieve a synergistic photothermal/chemo‐therapy. The as‐developed biomimetic nanocarriers, with their dual‐drug delivery features, homotypic tumor targeting and synergetic photothermal/chemo‐therapy, show much promise as a potential platform for cancer treatment.

## Graphical abstract

A biomimetic versatile nanocarrier is fabricated via the crosslinking reaction of hyaluronic acid with the ancillary anticarcinogen cisplatin in the presence of doxorubicin‐decorated gold nanorods followed by cloaking of cancer cell membrane. Once irradiated by NIR, in situ photothermal therapy is conducted which further induces hyperthermia‐triggered on‐demand drug release from the nanogel reservoir to achieve a synergistic photothermal/chemo‐therapy.

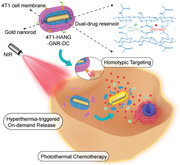

In the past decades, nanoparticle‐based drug delivery systems have received widespread attention for the treatment of various tumors.^[^ ^1^ ^]^ This interest was sparked by the fact that nanoparticles can achieve passive tumor targeting due to the enhanced permeability and retention effect present in a number of solid tumors, which would in principle allow a more effective chemotherapy.^[^ ^2^ ^]^ However, the therapeutic efficiency of nanodrugs is often disappointing and high recrudescence still remains an ongoing challenge.^[^ ^2^, ^3^ ^]^ In particular, single‐drug delivery systems inevitably result in severe systemic side effects because a large dose of drug is often required to achieve effective treatment.^[^ ^4^ ^]^ A possible solution to this problem is to develop synergetic nanotherapeutic systems with dual antitumor drugs and combination therapies to achieve high therapeutic efficiency with minimal side effects.

Recently, a biodegradable nanogel crosslinked by cisplatin (CDDP) and loaded with doxorubicin (DOX) was designed as a dual‐drug delivery system.^[^ ^5^ ^]^ Due to the combined action of DOX and CDDP on DNA, significant synergistic effects were achieved with this co‐delivery system, which improved the antitumor efficacy.^[^ ^6^ ^]^ However, a mere chemotherapy‐based treatment may not be fully adequate in all situations, which has led to the development of the concept of combination strategies. In this regard, besides chemotherapy, photothermal therapy has attracted increasing attention as a minimally invasive technique.^[^ ^7^ ^]^ It can convert NIR laser irradiation effectively into thermal energy by activating photothermal conversion agents, which induce the apoptosis of tumor cells through thermal ablation.^[^ ^8^ ^]^ Different types of photothermal conversion agents have been developed, such as carbon nanotubes,^[^ ^9^ ^]^ hollow copper sulfide nanoparticles,^[^ ^10^ ^]^ and gold nanorods (GNRs).^[^ ^11^ ^]^ Because of their facile surface modification, tunable optical–electronic properties, efficient photothermal conversion and high biocompatibility, GNRs have been widely applied for tumor ablation.^[^ ^12^ ^]^ Moreover, antitumor drugs have been conjugated covalently onto the surface of GNRs to enable the combination of chemo‐ and thermo‐therapy, although the limited GNR surface results in low drug loading quantity. In addition, the low targeting efficiency and short circulation time of GNRs in vivo also limit their application potential.

Nanocarriers camouflaged with isolated cell membranes have shown to be excellent candidates for cancer therapy recently.^[^ ^13^ ^]^ Cell membranes derived from various sources, such as red blood cells,^[^ ^14^ ^]^ platelets,^[^ ^15^ ^]^ macrophages,^[^ ^16^ ^]^ stem cells,^[^ ^17^ ^]^ and cancer cells^[^ ^18^ ^]^ were used to fabricate biomimetic nanoparticles for a range of applications. Cloaking nanoparticles with cancer cell membranes could bestow upon them the intrinsic ability of homologous targeting, increase the in vivo stability of the nanocarriers and even reduce drug leakage.^[^ ^19^ ^]^ This biomimetic technique has been employed to various materials (polymers^[^ ^20^ ^]^ and metals^[^ ^21^ ^]^) and shapes (spheres^[^ ^22^ ^]^ and cages^[^ ^23^ ^]^).

Herein, we report the design and construction of a biomimetic nanocarrier based on GNRs, which was coated with a cancer cell membrane for synergetic tumor photothermal and chemotherapy (**Scheme** **1**). The surface of the GNRs was first modified with hyaluronic acid (HA, Mn = 89 350 g mol^−1^), followed by the incorporation of DOX via electrostatic interactions, and CDDP via a chelating reaction, resulting in GNRs encapsulation in a stable CDDP‐crosslinked DOX‐loaded nanogel (HANG‐GNR‐DC). The nanogels were subsequently cloaked with the isolated cell membrane of 4T1 cells (4T1‐HANG‐GNR‐DC), in which Thomsen–Friedenreich (TF)‐antigen and CD47 were highly expressed on the surface. The membrane proteins, especially the TF‐antigen, play a key role in homotypic interactions among the 4T1 cells.^[^ ^24^ ^]^ With the dual stabilizing effect from the HA nanogel and the 4T1 cell membrane, this system displayed remarkably prolonged blood circulation and avoided premature release. The excellent homologous targeting ability obtained from the 4T1 cell membrane, together with the synergetic effect of photothermal (intrinsic photothermal conversion from GNRs) and chemotherapy (DOX‐CDDP) led to an outstanding performance of 4T1‐HANG‐GNR‐DC toward tumor in vivo.

**Scheme 1:**
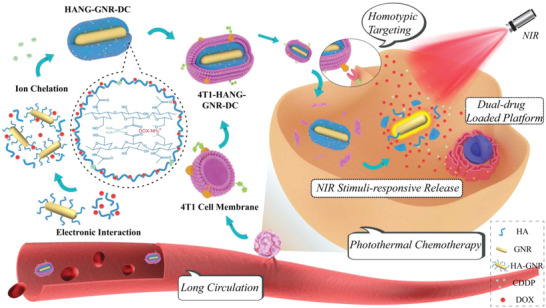
Schematic illustration of the fabrication of biomimetic DOX‐CDDP‐loaded GNRs and their application for synergistic chemotherapy and homotypic targeted photothermal therapy toward tumor cells.

GNRs were first synthesized based on seed‐mediated growth methods, using CTAB as stabilizing agent.^[^ ^25^ ^]^ According to UV–vis spectroscopy (**Figure** **1**A), the formed GNRs displayed two characteristic peaks including the longitudinal surface plasmon resonance (LSPR) peak at 810 nm and transverse surface plasmon resonance peak at 508 nm, suggesting the successful fabrication of rod‐shaped nanostructures. To replace the CTAB, end‐thiolated HA (HA‐SH) (Figure S1A, Supporting Information) was then synthesized and covalently attached onto the surface of GNRs, which facilitated the loading process of DOX together with additional HA via electrostatic interactions.^[^ ^26^ ^]^ Subsequently, CDDP was introduced dropwise into the mixture as physical crosslinker to obtain HANG‐GNR‐DC (dual‐drug encapsulation) due to the chelation of CDDP with the side carboxyl groups from HA.^[^ ^5^ ^]^ The nanoparticles were finally cloaked with cancer cell membrane vesicles (CCMs) harvested from 4T1 cells (4T1‐HANG‐GNR‐DC) via ultrasonication and physical co‐extrusion. The LSPR peak of the resulting nanostructure was red‐shifted to 818 nm, possibly because of the changed molecular conformation after coating with the cell membrane or HA.^[^ ^27^ ^]^ Besides, the characteristic peaks of DOX were observed in 4T1‐HANG‐GNR‐DC, indicating the integrity of the drug loaded HA nanogel after physical co‐extrusion (Figure 1B). The drug‐loading content and drug‐loading efficiency of 4T1‐HANG‐GNR‐DC were 8.4% and 46.7% for DOX, and 2.7% and 66.7% for CDDP, respectively.

**Figure 1:**
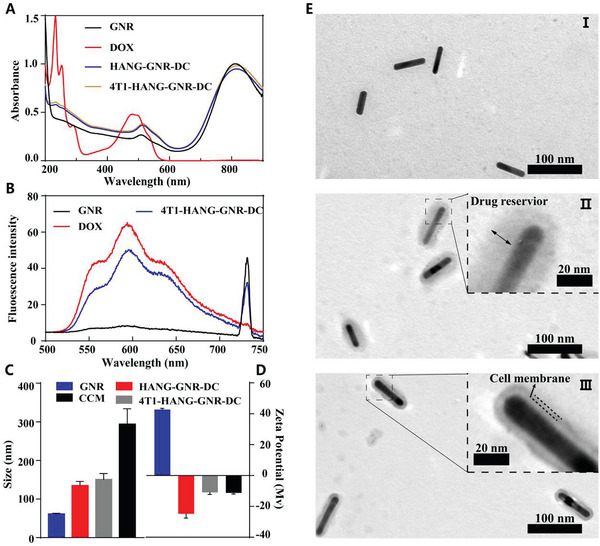
A) UV–vis spectra of GNR, DOX, HANG‐GNR‐DC, and 4T1‐HANG‐GNR‐DC. B) Fluorescence spectra of GNR, DOX, and 4T1‐HANG‐GNR‐DC with the excitation wavelength of 490 nm. C,D) Particle sizes intensity and zeta potentials of the GNR, HANG‐GNR‐DC, 4T1‐HANG‐GNR‐DC, and CCM vesicles measured by DLS. E) TEM images of GNR (I), HANG‐GNR‐DC (II), and 4T1‐HANG‐GNR‐DC (III).

The hydrodynamic size of the prepared HANG‐GNR‐DC nanoparticles (92.0 ± 9.4 nm) was larger than that of the pure GNRs (60.3 ± 8.6 nm) according to Zetaview measurements, which revealed that a HA nanogel of ≈30 nm thickness was introduced onto the GNRs (Figure S1B, Supporting Information). The presence of HA also led to a negative zeta potential of HANG‐GNR‐DC (−24.8 ± 2.8 mV) compared with the positively charged GNRs (42.6 ± 0.8 mV; Figure 1D). After cloaking with CCM vesicles, the size slightly increased to 103.1 ± 7.6 nm, while the surface displayed a charge (−10.2 ± 1.1 mV) which was similar to that of CCM vesicles (−11.3 ± 0.6 mV). All the structures were characterized subsequently by TEM. HANG‐GNR‐DC displayed a clear oval shape, consisting of the GNR as the inner core and the drugs‐loaded HA nanogel as the outer layer (Figure 1E‐II). Using ImageJ, the diameter distribution of HANG‐GNR‐DC was determined to be around 80–90 nm (Figure S1C–E, Supporting Information). It was noted that the thickness of the nanogel was relatively close to the elevated size of HANG‐GNR‐DC, indicating drugs, HA, and GNRs were crosslinked into a unified system. The TEM of 4T1‐HANG‐GNR‐DC (Figure 1E‐III) also provided visual evidence that a core (HANG‐GNR‐DC)‐shell (4T1 cell membrane) structure was formed with a cell membrane thickness of ≈5 nm.^[^ ^28^ ^]^ DLS was further used to analyze the size of GNR, HANG‐GNR‐DC, 4T1‐HANG‐GNR‐DC, and CCM vesicles, which showed a similar tendency as with the Zetaview measurements (Figure 1C). Additionally, the membrane proteins of 4T1‐HANG‐GNR‐DC were also analyzed using sodium dodecyl sulfate–polyacrylamide gel electrophoresis (SDS‐PAGE). It was observed that 4T1 cell membrane proteins were retained, demonstrating that biomimetic membranes were successfully coated onto the surface of the nanocarriers (Figure S2, Supporting Information).

After confirming the structure, the photothermal conversion properties were evaluated with a real‐time infrared thermal camera. The temperature of samples containing HA‐GNRs, HANG‐GNR‐DC, and 4T1‐HANG‐GNR‐DC sharply rose above 50 °C under laser irradiation for 480 s (808 nm, 1 W cm^−2^) while the temperature of PBS only increased 5 °C (**Figure** **2**A,B), indicating that nanogel formation and cytomembrane coating did not affect the photothermal effect. To investigate their photostability, four cycles of laser irradiation were applied to the 4T1‐HANG‐GNR‐DC, which all showed similar photothermal conversion (Figure 2C). The cellular internalization of 4T1‐HANG‐GNR‐DC was investigated with 4T1 cells (Figure S3, Supporting Information). When treated with HANG‐GNR‐DC, a much higher DOX fluorescence was observed inside the cells compared with free DOX, demonstrating the ability of the formed drug‐loaded nanogel to facilitate cellular uptake. After cloaking with the 4T1 cell membrane, the 4T1‐HANG‐GNR‐DC exhibited a dramatically increased fluorescence because the homotypic targeting ability derived from the active proteins of the outer cell membrane significantly improved the internalization of nanocarriers.

**Figure 2:**
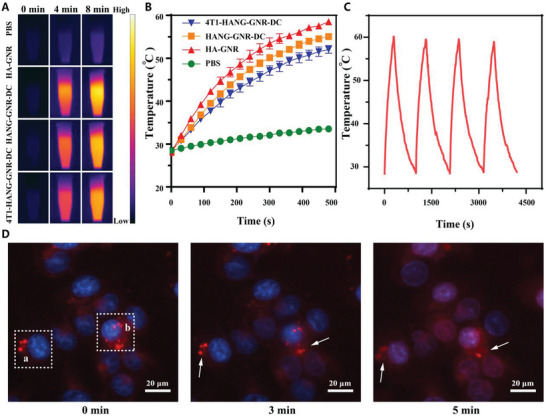
A) Thermal images and B) temperature increase curve of PBS, HA‐GNR, HANG‐GNR‐DC, and 4T1‐HANG‐GNR‐DC under 808 nm laser irradiation (1 W cm^−2^). C) Photostability of 4T1‐HANG‐GNR‐DC under laser irradiation. D) NIR‐triggered DOX release from internalized 4T1‐HANG‐GNR‐DC after irradiation of NIR laser at 0, 3, and 5 min (red = DOX, blue = nucleus).

To stimulate drug release from the particles after cellular uptake, NIR irradiation was employed.^[^ ^29^ ^]^ As shown in Figure 2D, the intracellular DOX release from 4T1‐HANG‐GNR‐DC was investigated in the homologous 4T1 cells. After endocytosis, dot‐like concentrated red fluorescence was observed. However, during laser irradiation for 5 min, the fluorescence diffused quickly, then dispersed and scattered around the cell nucleus, indicating effective release of DOX from 4T1‐HANG‐GNR‐DC. At the same time, the cell nuclei slightly turned round possibly because of the cell apoptosis caused by GNRs‐induced hyperthermia. Furthermore, two lines (a,b) were plotted along the fluorescent images to analyze the intensity changes of DOX with time. As shown in Figure S4A,C, Supporting Information, the peak 1 in both line a and b decreased sharply after 5 min irradiation, demonstrating the rapid release of DOX triggered by NIR and subsequent diffusion to the surrounding areas. At the meantime, the maximum value of the fluorescence intensity in region a and b (Figure 2D) was 1.8‐ and 3.5‐fold lower, respectively than that of the system without laser irradiation, while both of the regions retained a nearly identical mean fluorescence intensity before and after NIR treatment (Figure S4B,D, Supporting Information). This phenomenon was therefore attributed to NIR‐triggered drug release, rather than laser‐induced fluorescence quenching.

Three cell lines including 4T1 cells, melanoma cells (B16‐F10 cells), and African green monkey kidney cells (COS‐7 cells) were used to evaluate cell‐selective uptake of our nanoparticles (**Figure** **3**A). 4T1 cells labeled with DiO (green fluorescence) were co‐cultured with B16‐F10 cells. After incubating with 4T1‐HANG‐GNR‐DC for 6 h, the intracellular DOX fluorescence was obviously observed in 4T1 cells (white square in Figure 3A) while almost no fluorescence was found in B16‐F10 cells (white arrow). Flow cytometry (FCM) results (Figure 3B) confirmed that 4T1 cells possessed a higher intensity of the fluorescence signals originating from DiO and DOX, compared to that of B16‐F10 cells. The mean fluorescence intensity of DOX in 4T1 cells was 4.9‐fold higher than that of B16‐F10 cells (Figure 3C), indicating the efficient uptake of 4T1‐HANG‐GNR‐DC by the homologous cells. DiO labeled 4T1 cells co‐cultured with COS‐7 cells were also used to estimate the uptake difference between tumor and normal cells. As shown in Figure 3C, DOX fluorescence in 4T1 cells (white square) was 12.2‐fold higher than that in COS‐7 cells (white arrow in Figure 3A), which was also confirmed by the FCM measurement. Therefore, our 4T1‐HANG‐GNR‐DC nanoparticles can efficiently distinguish homologous cancer cells from normal tissue in vitro via highly specific self‐recognition. The red blood cells (RBCs) membrane was further used to demonstrate that the highly homologous targeting effect was derived from the 4T1 cell membranes cloaking. After incubating with 4T1 cells for 6 h, the intracellular DOX fluorescence could be observed obviously when treated with 4T1‐HANG‐GNR‐DC, while only slight fluorescence intensity for RBC‐HANG‐GNR‐DC. The results indicated that camouflaging with 4T1 cell membrane provided the much higher cellular uptake in homotypic tumor cells than that with non‐tumor cells membranes.

**Figure 3:**
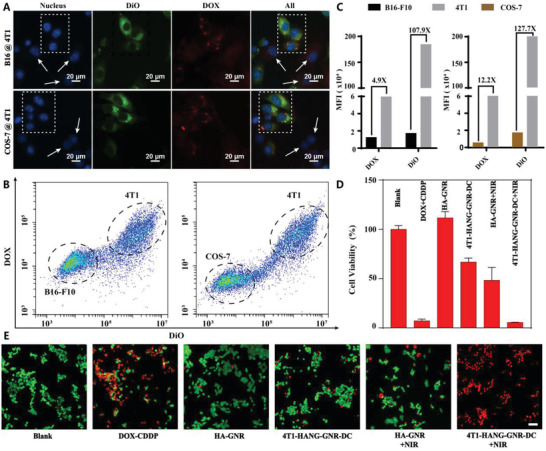
A) 4T1‐HANG‐GNR‐DC incubation with co‐cultured 4T1/B16‐F10 cells and 4T1/COS‐7 cells (red = DOX, green = 4T1 cells membrane, blue = nucleus). B) Flow cytometric profiles and C) mean fluorescence intensity of the co‐cultured 4T1/B16‐F10 cells and 4T1/COS‐7 cells upon 6 h incubation with 4T1‐HANG‐GNR‐DC. D) Viability of 4T1 cells with different treatments. E) Fluorescence images of live/dead staining of 4T1 cells with different treatments. The live cells were stained with AM (green) and the dead cells were stained with PI (red). Scale bars = 50 µm.

The antiproliferative effects and dark cytotoxicity on 4T1 cells were investigated with an MTT assay. As shown in Figure 3D, no cytotoxicity was observed when the cells were treated with HA‐GNR, indicating the biocompatibility of the GNR. After laser irradiation, the viability of 4T1 cells decreased to 49% because of the photothermal effect induced by GNR. For our 4T1‐HANG‐GNR‐DC without laser treatment, 33% of the 4T1 cells were killed due to homologous targeting and co‐delivery of the loaded DOX and CDDP. More than 94% cells were dead after incubation with 4T1‐HANG‐GNR‐DC followed by NIR irradiation, which could be attributed to the combined effect of photothermal and chemotherapy (thermal ablation of cancer cells and the rapid dual‐drug release triggered by hyperthermia). The live (green)/dead (red) staining was further used to evaluate the in vitro phototoxicity, which was consistent with the MTT results (Figure 3E). Upon NIR irradiation, almost all the cells treated with 4T1‐HANG‐GNR‐DC were dead and displayed the most intensive red fluorescence.

After confirming the in vitro performance of our design, in vivo photothermal imaging was conducted after administration of normal saline (NS), HA‐GNR, HANG‐GNR‐DC, and 4T1‐HANG‐GNR‐DC to tumor‐bearing mice for 24 h (**Figure** **4**A). As shown in Figure 4B, tumor‐bearing mice treated respectively with NS, HA‐GNR, and HANG‐GNR‐DC displayed a slight temperature increase during irradiation for 2 min (808 nm, 1.0 W cm^−2^) probably because of the absorbance of NIR by tissues and low accumulation of GNR in tumor area. When injected with 4T1‐HANG‐GNR‐DC, the local temperature rapidly increased to 64 °C within 2 min after irradiation (Figure 4B). This demonstrated high tumor targeting and outstanding heat generating efficiency of 4T1‐HANG‐GNR‐DC.

**Figure 4:**
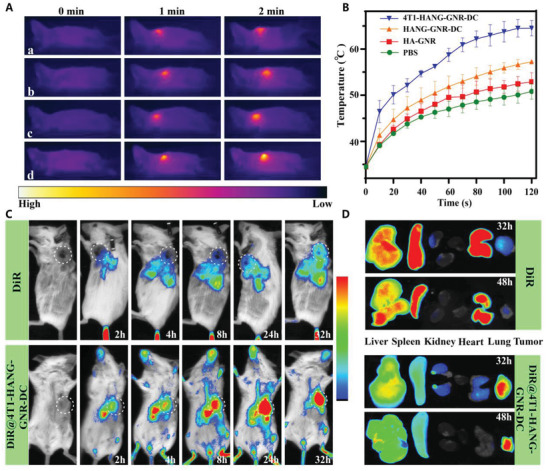
A) Infrared thermography of tumor‐bearing mice injected with a: NS, b: HA‐GNR, c: HANG‐GNR‐DC, and d: 4T1‐HANG‐GNR‐DC under irradiation for 2 min (808 nm, 1.0 W cm^−2^). B) Temperature curve of the tumor from the mice during NIR irradiation. C) In vivo distribution of free DiR and DiR@4T1‐HANG‐GNR‐DC in tumor‐bearing mice at different time points. D) Ex vivo tissue distribution of sacrificed mice at 32 and 48 h after the injection.

To further assess the tumor targeting ability and biodistribution, a near‐infrared fluorescent dye (DiR) was introduced into the lipid bilayer of coated 4T1 cell membrane for in vivo imaging. Free DiR and DiR@4T1‐HANG‐GNR‐DC with the same DiR concentration of 594 µg mL^−1^ were administrated intravenously into tumor‐bearing mice and in vivo imaging was recorded at different time intervals with an in vivo fluorescence imaging system. After injecting with DiR@4T1‐HANG‐GNR‐DC, the fluorescence in the tumor region gradually increased (Figure 4C; Figure S6A, Supporting Information), indicating the efficient tumor homing of our DiR@4T1‐HANG‐GNR‐DC, while for free DiR, most of the fluorescence was widely distributed in the main organs and only a slight signal was observed at the tumor site after 24 h. The relative fluorescence intensity of DiR@4T1‐HANG‐GNR‐DC in the tumor region was 3.7‐fold higher than that of free DiR at 32 h (Figure S6A, Supporting Information), showing excellent homotypic targeting ability of the cell membrane coated nanoparticles. After 32/48 h of injections, the mice were sacrificed and the main organs (liver, heart, spleen, lung, kidney, and tumor) were collected to analyze their detailed biodistribution profiles (Figure 4D). For free DiR, the fluorescence mainly concentrated in the metabolic organs (such as liver, spleen) and lung with limited accumulation in the tumor. As comparison, the tumor exhibited the highest fluorescence signal when DiR@4T1‐HANG‐GNR‐DC were administrated, indicating the advantage of the homotypic membrane cloaking strategy for tumor specific accumulation (Figure 4D; Figure S4B, Supporting Information). Of note, the fluorescence signal in the tumor decreased only 7.6% from 32 to 48 h, which demonstrated a prolonged retention in the tumor region.

The excellent homologous targeting capacity of 4T1‐HANG‐GNR‐DC also resulted in outstanding local photothermal effect. Induced hyperthermia may not only cause the thermal ablation of cancer cells, but also stimulate the immune cells, such as macrophages and dendritic cells (DCs). To test the immune response of our biomimetic nanoparticles, macrophages (RAW 264.7) were used to study their migration capacity after different treatments (Figure S7, Supporting Information). Interestingly, most macrophages migrated toward the group incubated with 4T1‐HANG‐GNR‐DC followed by NIR irradiation. The results showed that our system could shape a stronger chemotaxis environment and trigger the positive response of macrophages, which might attribute to the production of apoptosis bodies accompany with the “eat me” signal.^[^ ^30^ ^]^ Besides, we further investigated the maturation of DCs after hyperthermia therapy. An obvious effect on promoting the maturation of the DCs was also observed after treatment (Figure S8, Supporting Information). Therefore, we believed that our biomimetic nanocarriers could play a positive role in stimulation of the immune cells.

Encouraged by the efficient accumulation of 4T1‐HANG‐GNR‐DC in the tumor site, we then assessed the in vivo antitumor efficacy with BALB/c mice (female, 6 weeks) bearing a 4T1 xenograft mammary tumor. The mice were divided into seven groups (*n* = 4): 1) NS; 2) 4T1‐HANG‐GNR‐DC; 3) HANG‐GNR‐DC; 4) DOX‐CDDP; 5) HANG‐GNR‐DC + NIR; 6) HA‐GNR + NIR; 7) 4T1‐HANG‐GNR‐DC + NIR. The tumor regions were irradiated for 2 min with a NIR laser (808 nm, 1 W cm^−2^), 24 h after administration on day 2 and day 8. The tumor sizes were recorded for 16 days, after which the tumors were collected and weighted (**Figure** **5**A,B). As expected, 4T1‐HANG‐GNR‐DC showed better tumor inhibition than HANG‐GNR‐DC, indicating the advantages of homotypic targeting of our biomimetic nanoparticles (Figure 5B). When irradiated with the 808 nm laser after intravenous injection of 4T1‐HANG‐GNR‐DC, the mice gained the best tumor inhibition effect and the tumor volume was suppressed by 96% compared with the saline group, which was even lower than the initial tumor volume before treatment (Figure 5A; Figure S5A, Supporting Information). The body weights of the mice during the treatment showed no significant decrease, suggesting no acute toxicity of 4T1‐HANG‐GNR‐DC (Figure 5C). Serum biochemical parameters including alanine aminotransferase, aspartate aminotransferase, glutamyl transpeptidase, and total bilirubin as liver function indicators, as well as kidney function indicators of blood urea nitrogen (UREA) and creatinine were further determined, demonstrating good biocompatibility and negligible systemic toxicity of the biomimetic nanocarriers (Figure S9B, Supporting Information). After 16 days, the mice were sacrificed and the tumors were collected. As shown in Figure 5D, the tumor volumes of two mice treated with 4T1‐HANG‐GNR‐DC + NIR decreased to almost zero. As expected, the synergistic effect of photothermal therapy and laser‐induced rapid chemotherapy dramatically improved the anti‐tumor performance of the nanocarriers. Two tumor‐bearing mice cured respectively by 4T1‐HANG‐GNR‐DC + NIR and HANG‐GNR‐DC + NIR were selected for further long‐term observation. Interestingly, tumor recurrence was observed for HANG‐GNR‐DC + NIR treatment while full remission was noticed in the 4T1‐HANG‐GNR‐DC + NIR group (Figure 5E). Therefore, 4T1 cancer cell membrane cloaking does not only improve the direct therapeutic effect, but also prevents tumor recurrence.

**Figure 5:**
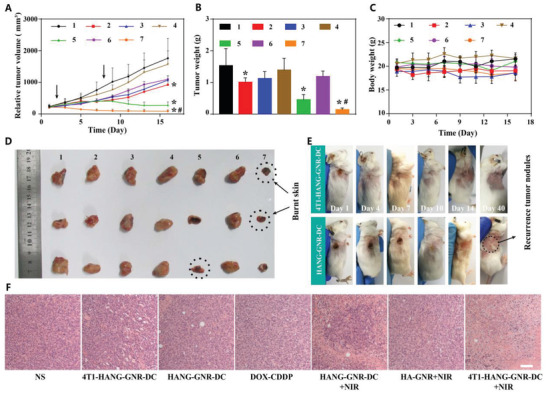
A) Tumor volume of 4T1 tumor‐bearing mice treated with different preparations (1, NS; 2, 4T1‐HANG‐GNR‐DC; 3, HANG‐GNR‐DC; 4, DOX‐CDDP; 5, HANG‐GNR‐DC + NIR; 6, HA‐GNR + NIR; 7, 4T1‐HANG‐GNR‐DC + NIR) during 16 days treatment. B) Tumor weight and C) body weight of the mice from different groups. D) Collected tumors from each group. E) Observation of tumor recurrence of mice after 16 days anti‐tumor treatment. F) H&E staining of the tumor tissues after different treatments (scale bars = 20 µm). Mean ± SD (*n* = 4), *p < 0.05 (compared with the NS group), ^#^ *p* < 0.05 (compared with the HANG‐GNR‐DC + NIR group).

Furthermore, H&E staining was carried out after treatment. It was shown that almost all the cells were apoptotic/dead after treatment with 4T1‐HANG‐GNR‐DC + NIR, indicating a significant tumor targeting effect (Figure 5F). The H&E staining of the main organs was also used to estimate the long‐term toxicity of all the groups. No apparent morphological changes were observed, which showed again the good biocompatibility of 4T1‐HANG‐GNR‐DC with NIR irradiation (Figure S10, Supporting Information).

In summary, we have successfully demonstrated the fabrication of a biomimetic versatile nanocarrier (4T1‐HANG‐GNR‐DC) for a highly efficient and selective synergistic chemo/photothermal therapy to overcome single‐drug resistance and tumor recurrence. Antitumor drugs DOX and CDDP together with hyaluronic acid were co‐loaded onto the surface of GNRs as nanogels via electrostatic interactions, followed by cloaking of the nanogel with a cancer cell membrane. Due to the homologous targeting effect, the resulting 4T1‐HANG‐GNR‐DC nanoparticles accumulated efficiently and possessed a long‐time retention in the tumor region. The high photothermal conversion efficiency of GNR was used to perform in situ photothermal therapy under NIR irradiation and the produced heat also provided a trigger for on‐demand drug release in vivo. Our work thus presents a smart strategy to combine photothermal tumor ablation and controlled chemotherapy using a biomimetic nanocarrier to achieve a synergistic therapeutic outcome. Taken together, the proposed formulation may serve as a promising platform for combined photothermal and chemotherapy; it could furthermore be easily extended to the controlled delivery of cationic drugs or biomacromolecules, such as proteins and antibodies, to improve the therapeutic potential further.

## Experimental Section

##### Preparation of HA‐GNR Cores

Thiol modified hyaluronic acid (HA‐SH) was first synthesized by reductive amidation.^[^ ^26^ ^]^ HA (50 mg) and cystamine dihydrochloride (30 mg) were dissolved in borate buffer (0.1 m, pH = 8.5) containing 0.4 m sodium chloride (10 mL) and the resulting solution was stirred for 2 h. Then sodium cyanoborohydride (126 mg) was added to the above solution at 40 °C. After 5 days, 100 mm DTT was added and the solution was stirred for 12 h. Then the mixture was dialyzed against with sodium chloride solution (100 mm), 25% ethanol, and distilled water in sequence for a day, followed with lyophilization. Before use, HA‐SH was treated with TCEP for the activation of sulfhydryl group. Then the GNRs solution (0.37 mg mL^−1^, 3 mL) was reacted with activated HA‐SH (1 mg mL^−1^, 150 µL) for 6 h to form HA‐GNR. HA‐GNR solution was centrifuged (6500 g, 30 min, 20 °C) to remove excess HA‐SH, and resuspended in distilled water.

##### Preparation of HANG‐GNR‐DC

HANG‐GNR‐DC was synthesized according to in situ crosslinking process.^[^ ^5^ ^]^ HA (1.5 mL, 1 mg mL^−1^) and DOX (0.18 mL, 3 mg mL^−1^) were added into the HA‐GNR and the resulting solution was reacted for 12 h under dark (800 rpm). CDDP dissolved in DI water (327 µL, 0.2737 mg mL^−1^) was slowly dropped into the mixture at the speed of 40 µL min^−1^. After stirring (800 rpm) for 3 days, the mixture was centrifuged (6500 g, 20 min, 20 °C) for the purification of the dual‐drug loading nanocarriers (HANG‐GNR‐DC) and redispersed in PBS (0.1 m, pH = 7.4).

##### Preparation of 4T1‐HANG‐GNR‐DC

To obtain the 4T1 CCMs, murine mammary carcinoma cells were centrifuged for 5 min (3000 r min^−1^, 4 °C) to collect pure precipitations and then resuspended in hypotonic lysing buffer (containing membrane protein extraction reagent and PMSF).^[^ ^31^ ^]^ After incubating with an ice bath for 12 min, 4T1 cells were transferred into Dounce homogenizer for mechanical disruption with 40 times. Afterward, the mixtures were centrifuged with 700 g at 4 °C for 12 min and the resulting supernatant was centrifuged again with 14 000 g for 40 min. The final 4T1 cell membrane fragments were collected and lyophilized for quantification and storage (−20 °C). Before use, the fragments (1 mg) were redispersed in PBS (0.1 m, pH = 7.4) and sonicated for 10 s, and then extruded through 800 and 400 nm polycarbonate membranes (LiposoFast, Avestin, Canada) at least five times respectively to obtain 4T1 CCMs. HANG‐GNR‐DC were then mixed with 4T1 CCMs (1 mg) solution to co‐extrude via 400 nm polycarbonate membranes at least for five circles. Then, the mixture was centrifuged with 5000 g at 4 °C for 20 min to achieve final formulation (4T1‐HANG‐GNR‐DC).

##### NIR Triggered Intracellular On‐Demand Release of DOX

4T1 cells were seeded onto 6‐well plate with the density of 2 × 10^5^ cells per well and cultured for 24 h. 4T1‐HANG‐GNR‐DC (800 µg mL^−1^) in serum‐free medium was added and incubated for 4h. After replacing with fresh serum‐free medium, the resulting 4T1 cells were treated with laser irradiation (808 nm, 1W cm^−2^) for 5 min. The fluorescence images of the cells were recorded at desired time points.

##### Homologous‐Targeting 4T1 Cell Line In Vitro

B16‐F10 (or COS‐7) and DiO‐labeled 4T1 cells (labeling with DiO for 30 min before digestion) were co‐cultured in 24‐well plate with the density of 2.5 × 10^4^ per well respectively for 24 h. Then the medium was replaced by 4T1‐HANG‐GNR‐DC (800 µg mL^−1^ in serum‐free medium). After incubating for 6 h, the nucleus was labeled with DAPI, and all the groups were observed with fluorescence microscope. For FCM, B16‐F10 (or COS‐7) and DiO‐labeled 4T1 cells were performed with the same procedure mentioned above. The cells were collected and detected by FCM.

Materials and instruments, other experimental details and additional figures are arranged in Supporting Information. All the animal procedures were carried out under the guideline approved by the Institutional Animal Care and Use Committee (IACUC) of Southern Medical University (permit number: SYXK 2016‐0167).

## Conflict of Interest

The authors declare no conflict of interest.

## Author Contributions

J.G. and Y.T. conceived and designed the experiments. J.G., F.W., S.W., L.L., K.L., Y.Y., Z.W., H.W., B.C., J.J., and J.O. carried out the experiments. J.G. wrote the first draft of the manuscript with contributions from all co‐authors.

## Supporting information

Supporting Information
