# Supplementary material for: Hyperthermia‐Triggered On‐Demand Biomimetic Nanocarriers for Synergetic Photothermal and Chemotherapy
Source: Adv Sci (Weinh). 2020 Apr 20;7(11):1903642. doi: 10.1002/advs.201903642 (PMC7284223; doi:10.1002/advs.201903642)
Supplement: Supplementary file 1 — Supporting Information [file ADVS-7-1903642-s001.pdf]

Copyright WILEY-VCH Verlag GmbH & Co. KGaA, 69469 Weinheim, Germany, 2020.

Supporting Information

Hyperthermia-triggered On-demand Biomimetic Nanocarriers for Synergetic Photothermal and Chemotherapy

Junbin Gao, Fei Wang, Shuanghu Wang, Lu Liu, Kun Liu, Yicheng Ye, Zhen Wang, Hong Wang, Bin Chen, Jiamiao Jiang, Juanfeng Ou, Jan C. M. van Hest, Fei Peng* and Yingfeng Tu**

Contents

1. Materials and instruments..2

2. Preparation of Gold nanorods..2

3. Cell Membrane Proteins Analysis..3

4. DOX and CDDP Loading Capacity..3

5. *In Vitro* Photothermal Performance..4

6. Cell Culture and Cell Uptake..4

7. *In Vitro* Cell Viability..5

8. Animal Procedures..5

9. *In Vivo* Temperature Measurement on Tumor region..5

10. *In Vivo* and *Ex Vivo* Imaging and Biodistribution..6

11. *In Vitro* Response of Immune Cells under Hyperthermia Therapy..6

12. *In Vivo* Tumor Growth Inhibition and Safety Analyses..7

13. Statistical Analyses..8

1. Materials and instruments

Ascorbic acid, chloroauric acid ($HAuCl_4$), sodium borohydride, cetrimonium bromide (CTAB), sodium cyanoborohydride, DL-dithiothreitol (DTT), MTT, dimethyl sulfoxide (DMSO) and hydrochloric acid were obtained from Sigma-Aldrich (St. Louis). Low molecular weight sodium hyaluronate was bought from Bloomage Freda Biopharm. Doxorubicin hydrochloride (DOX), cystamine dihydrochloride, silver nitrate ($AgNO_3$), tris(2-Carboxyethyl) phosphine hydrochloride (TCEP) and cisplatin (CDDP) were bought from Innochem. Hoechst 33342, DiO, DiR and Membrane and Cytosol Protein Extraction Kit were purchased from Beyotime Biotechnology. Transwell 3422 and 3472 were brought from the Coring Incorporated. BCA protein assay kit was obtained from the Cusabio Biotech. The CD11c-FITC, CD80-PE and CD86-APC monoclonal antibody were purchased by the eBioscience. The All reagents for cell culture were bought from Gibco. The purified deionized water was prepared by the Milli-Q plus system (Millipore, USA).

The structure of the formed nanoparticles was analyzed with a JEM 1400 Transmission Electron Microscope (TEM) with an acceleration voltage of 120 kV (JEOL, Japan). Zeta potential and size measurements were performed on a Zetasizer Nano ZS (Malvern, UK). Hydrodynamic diameters were investigated with Nanoparticle Tracking Analysis (NTA) by Zetaview (PMX, Germany). The UV-vis absorption was measured with an UV-2600 spectrophotometer (SHIMADZU, Japan). Fluorescence spectra were analyzed with a RF-6000 fluorescence spectrophotometer (SHIMADZU, Japan). Cell morphologies were captured on a Ti2-A Inversion Fluorescence Microscope (Nikon, Japan). The mean fluorescence intensity of cells was analyzed with cytoFLEX (BECKMEN, USA). The thermographic images were acquired by an infrared thermal camera (FLIR, USA). Photothermal therapy on cells or mice were carried out with a 808 nm laser (Stone, China). All the parameters of blood biochemistry were analyzed by Pointcare M3 (MNCHIP, China). The *in vivo* imaging was performed by In-

Vivo FX PRO (BRUKER, Germany). The fluorescence image of cell uptake was performed by the CLSM of FLUOVIEW FV1000 (OLYMPUS, Japan)

2. Preparation of Gold nanorods

Seed-mediated growth methods was used for the synthesis of GNRs.[1] The freshly prepared ice-cold sodium borohydride solution (10 mM, 0.6 mL) was added into the seed solution containing CTAB (0.1 M, 10 mL) and $HAuCl_4$ (25.4 mM, 0.1 mL) and the resulting solution was stirred vigorously (1200 rpm) at 32 °C, followed with aging for 2 h. CTAB (0.2 M, 25 mL), $HAuCl_4$ (25.4 mM, 1 mL), Ascorbic acid (90 μM, 0.5 mL), $AgNO_3$ (12 mM, 0.45 mL) and HCl (1 M, 0.95 mL) were dissolved in distilled water (22.1 mL), which was used as growth solution. The seed solution (0.12 mL) was added into the above growth solution for 2 h incubation at 32 °C thermostatically. The formed GNRs were centrifuged (6500 g, 30 min, 20 °C) to remove excess CTAB and then redispersed in the distilled water.

3. Cell Membrane Proteins Analysis

The SDS-PAGE analysis was performed to estimate the retained proteins of the 4T1 cell membrane vesicles, HANG-GNR-DC and 4T1-HANG-GNR-DC. Briefly, equal amounts of proteins from different samples (quantitafied with BCA protein assay kit) were used for SDS-PAGE analysis. The gel was treated with Coommassie brilliant bule for stained and imaged. RBCs membranes and RBC-HANG-GNR-DC were also analyzed according to the same method.

4. DOX and CDDP Loading Capacity

The prepared 4T1-HANG-GNR-DC were centrifuged (6500 g, 20 min, 20 °C) and the supernatant containing free DOX and CDDP was collected for further quantification. To quantify the loading efficiency, the lyophilized 4T1-HANG-GNR-DC were then weighed precisely. UV-Vis spectrometer and ICP-OES were used to measure the amount of the DOX and CDDP. The LE/LC of DOX and CDDP were calculated respectively.

5. *In Vitro* Photothermal Performance

The formulations of HA-GNR, HANG-GNR-DC, 4T1-HANG-GNR-DC with same concentration of Au (85 μg/mL) were added into EP tube. All the samples were treated with NIR laser (808 nm, 1 W/cm^2) for 8 min, following with infrared imaging at each 30 s. To study the photostability of the resulting materials, 4T1-HANG-GNR-DC (800 μg/mL) were irradiated with NIR laser (808 nm, 1 W/cm^2) for 4 cycles, followed by infrared imaging recording.

6. Cell Culture and Cell Uptake

The 4T1 cell was used in this work and cultured in RPMI 1640 supplemented with 10% FBS, penicillin (50.0 IU mL^{-1}) and streptomycin (50.0 IU mL^{-1}). The cells were incubated at 37 °C in a atmosphere with 5% CO_2.

The cells were seeded in the 24-well plates with a density of 5 $\times 10^5$ per well and cultured for 24 h. Then the original medium was replaced by free DOX, HANG-GNR-DC or 4T1-HANG-GNR-DC solution in RPMI 1640 with a same DOX concentration of 50 μg/mL. After incubation for 8 h, the cells were washed and stained with Hoechst 33342 (blue) for 12 min, and all the groups were observed with fluorescence microscope.

To evaluated the uniqueness of the homologous targeting ability of the 4T1 cancer cell membranes, the non-tumor cell (RBCs) were isolated from the femal BALB/c mice. The whole blood was centrifuged at 3000 rpm for 5 min and then washed with saline for three times. The obtained RBCs were then re-suspended in DI water to induce membrane rupture. Subsequently, the solution was centrifuged at 13500 rpm for 8 min at 4 °C. The collected RBCs membranes were co-extruded with HANG-GNR-DC through the 400 nm polycarbonate film to obtained RBC-HANG-GNR-DC.

The 4T1 cells were seed in the 24-well plates with a density of 5 $\times 10^5$ per well and cultured for 24 h. Then the original medium was replaced repectively by 4T1-HANG-GNR-DC and RBC-HANG-GNR-DC solution in basic medium with the concentration of 400 μg/mL. After

incubation for 3 h and 6 h, the cell were washed and stained with Hoechst 33342 (blue) for 12 min, and all the groups were observed with the confocal fluorescence microscope.

7. *In Vitro* Cell Viability

4T1 cells were seeded onto 96-well plate with the density of 5×10^3 cells per well and cultured for 24 h. The media were replaced with fresh ones containing DOX-CDDP (50 μg/mL and 16 μg/mL), HA-GNR (200 μg/mL), 4T1-HANG-GNR-DC (with GNRs concentration at 200 μg/mL) or PBS (0.1 M, pH=7.4) respectively. After 6 h incubation, the cells were washed gently by fresh medium and then irradiated with or without 808 nm NIR laser for 5 min (1 W/cm^2). The cells were further incubated for 16 h, and the media were replaced by MTT solution (0.5 mg/mL in medium) and incubated for another 4 h. Subsequently, the media were removed and 150 μL DMSO was added into each well. To calculate the cell viability, the absorbance at 490 nm was measured by microplate reader (Biotek 800 TS, USA).

Live-dead staining was also used to study the *in vitro* anti-tumor efficacy of 4T1-HANG-GNR-DC. The 4T1 cells were seeded in 96-well plate and different formulations were added followed by NIR-irradiation. The resulting cells were stained with calcein-AM/PI for 30 min and observed on fluorescence microscope.

8. Animal Procedures

The female BALB/c mice were obtained from the Laboratory Animal Center of Southern Medical University, and used at age of 6 weeks. The tumor-bearing mice were prepared by subcutaneous injection in the armpits of right anterior limbs with 0.15 mL of cell suspension containing 1.5×10^6 murine mammary carcinoma 4T1 cells. All the animal procedures were carried out under the guideline approved by the Institutional Animal Care and Use Committee (IACUC) of Southern Medical University (permit number: SYXK 2016-0167).

9. *In Vivo* Temperature Measurement on Tumor region

The *in vivo* photothermal effects were also determined in the orthotopic breast tumor model. The femal BALB/c mice were injected subcutaneously with cell suspension (1.5×10^6 4T1 cells). After two weeks, the tumor bearing mice were injected intravenously with PBS, HA-GNR, HANG-GNR-DC and 4T1-HANG-GNR-DC (the GNR concentration was 10 mg/kg per mouse) respectively. After 24 h, the mice were anesthetized and the tumors were exposed to 808 nm laser at 1 W/cm^2 for 2 min, followed by near-infrared thermal imaging.

10. *In Vivo* and *Ex Vivo* Imaging and Biodistribution

For *in vivo* imaging, DiR (100 μL, 5 mM) was added into the solution of 4T1-HANG-GNR-DC (1 mg/mL) for 30 min to obtain DiR@4T1-HANG-GNR-DC. Then 4T1 tumor bearing BALB/c mice (n = 4) were injected intravenously with 200 μL free DiR and DiR@4T1-HANG-GNR-DC in PBS respectively with the same concentration of DiR (594 μg/mL). After 24 h, all mice were gaseous anesthetized and the *in vivo* fluorescence imaging was recorded by small animal imaging system (excitation/emission=920/980 nm). Then, all mice were sacrificed and their tumors, hearts, liver, spleens, lungs, kidneys were collected for *ex vivo* signals acquiring.

11. *In Vitro* response of immune cells under antitumor hyperthermia therapy

4T1 cells were seeded in the 24-well plates with a density of 5×10^5 cells per well and cultured for 24 h. Then the original medium was replaced repectively by PBS, HA-GNR, HANG-GNR-DC and 4T1-HANG-GNR-DC solution in basic medium with the same gold concentration of 150 μg/mL. Macrophages (RAW 264.7) with a density of 1×10^6 cells per mL, was seeded on the Transwell insert with 100 μL of the cell's suspension. After 12 h incubation, the 4T1 cells were washed with fresh 1640 medium and then irradiated with the NIR laser (808 nm, 1 W/cm^2) for 8 min. The Transwell inserts were placed subsequently onto the 4T1 cells cultured plates to observed the migration of macrophages. After co-culturing for 10 h, non-migrated cells in upper layer by the wet cotton swab were removed. The inserts

were then soaked in the 4 % paraformaldehyde for fixation and finally stained with 0.1 % gentian violet for imaging.

The dendritic cells were harvested and cultured according to the previous reports. [2] On day 6, the semi-mature DCs were collected and seeded on the 24-well plates with a density of 8 $\times 10^5$ cells per well and cultured for 24 h. The 4T1 cells were seeded onto the Transwell inserts with 2×10^5 cells per well. After 18 h, the medium of 4T1 cells were replaced by PBS, HA-GNR, HANG-GNR-DC and 4T1-HANG-GNR-DC solution (100 μL) in basic medium with the same gold concentration of 150 μg/mL, followed by 6 h incubation. After that, the cells were washed with fresh 1640 medium and then irradiated with the NIR laser (808 nm, 1 W/cm^2) for 8 min. Finally, the Transwell inserts were then placed to the DCs cultured plates to induce the maturation. After 12 h, the DCs with different treatments were centrifugated at 1500 rpm for 5 min respectively and then re-suspended in the HBSS containing 10 % FBS to stain with fluorescent antibody (CD11c-FITC, CD80-PE and CD86-APC) for followed flow cytometry analysis.

12. *In Vivo* Tumor Growth Inhibition and Safety Analyses

To perform the *in vivo* tumor therapy, the BALB/c mice with subcutaneous 4T1 xenografts (grew up to approximately 150-200 mm^3) were divided into seven groups according to the different treatments. (n = 4 per group): (1) NS, (2) 4T1-HANG-GNR-DC, (3) HANG-GNR-DC, (4) DOX-CDDP, (5) HANG-GNR-DC + NIR laser, (6) HA-GNR + NIR laser, (7) 4T1-HANG-GNR-DC + NIR laser. The concentration of GNR used was 10 mg/kg and the concentrations of DOX and CDDP were 0.83 and 0.27 mg/kg respectively. For NIR laser irradiation, the mice were irradiated for 2 min with a 808 nm laser (1W/cm^2) after injecting intravenously for 24 h. The tumor volume and mice body weight were measured every two days. After 16 days, the mice were euthanized and serum samples were separated. Biochemical parameters including aspartate aminotransferase (AST), alanine aminotransferase (ALT), Glutamyl transpeptidase (GGT), total bilirubin (TBIL), blood urea

nitrogen (UREA), creatinine (CRE), were analyzed automatically using Pointcare M3. The main organs including the liver, spleen, kidney, heart, and lung were collected for further hematoxylin-eosin (H&E) staining. To investigate the tumor recurrence, the mice after treatments were selected from the group of 4T1-HANG-GNR-DC or HANG-GNR-DC (the tumor disappeared after treatment) and observed for another 40 days.

13. Statistical Analyses

Data analyses were conducted using the software GraphPad Prism 7.0. The mean ± SD were determined for all the treatment groups. Statistical analysis was performed by Student's t-test (two-tailed). $P < 0.05$ was considered representative of a statistically significant difference between two groups.

References

[1] H. Lee, J. H. Lee, J. Kim, J. H. Mun, J. Chung, H. Koo, C. Kim, S. H. Yun, S. K. Hahn, *ACS Appl. Mater. Interfaces* **2016,** *8*, 32202.

[2]Y. Dong, A. A. Arif, G. F. Poon, B. Hardman, M. Dosanjh, P. Johnson, *JoVE (Journal of Visualized Experiments)* **2016**, e54194.

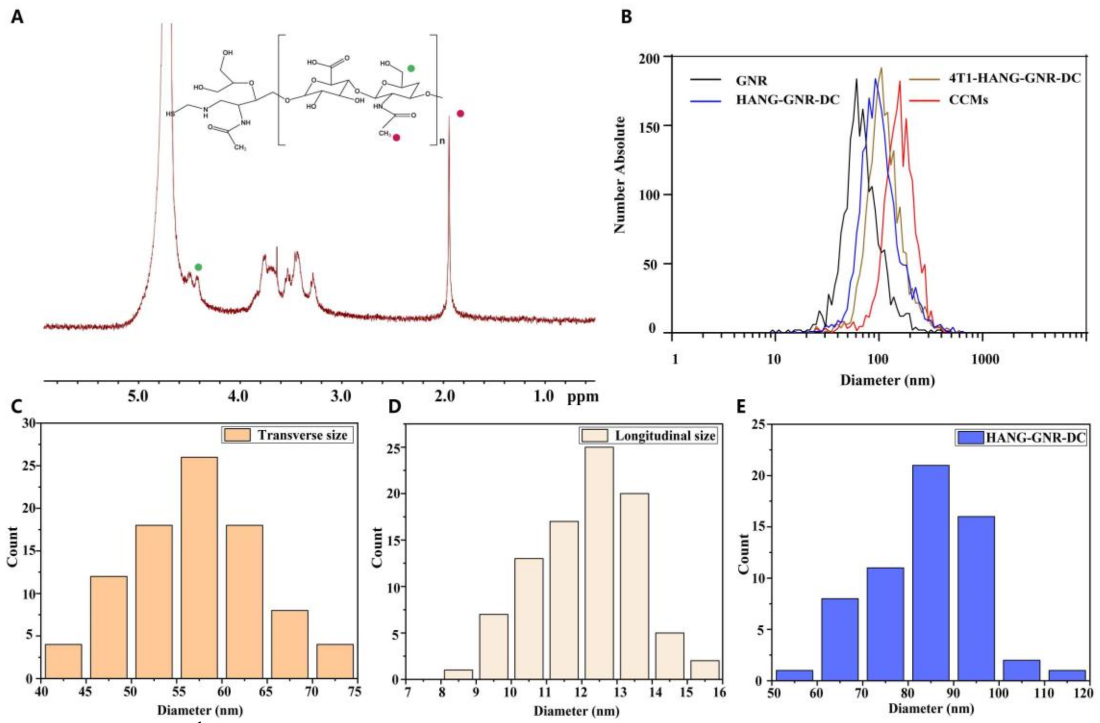

Figure S1. A) ^{1}H NMR measurement of thiolated HA (HA-SH) in D_2O. B) Hydrodynamic size distribution of GNR, HANG-GNR-DC, 4T1-HANG-GNR-DC and CCM obtained from ZetaView. C-D) Transverse size and longitudinal size distribution of GNR (Over 90 GNR were counted for statistical analysis). E) Diameter distribution of HANG-GNR-DC (Over 50 HANG-GNR-DC were counted for statistical analysis). The sizes of nanoparticles were collected from TEM images.

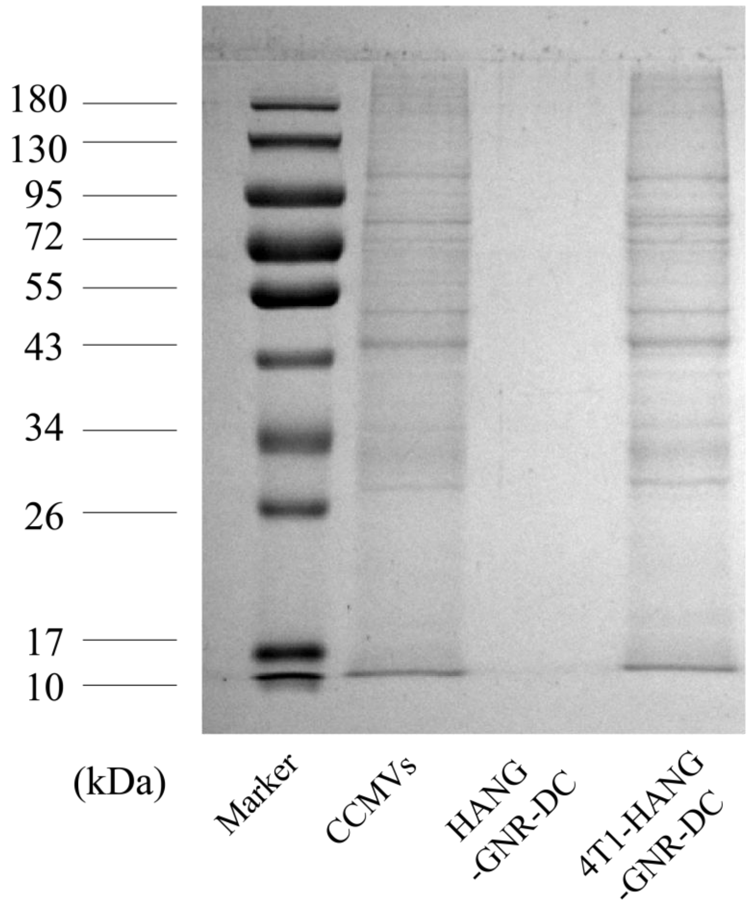

Figure S2. SDS-PAGE analysis of marker, CCMVs (4T1 cancer cell membrane vesicles), HANG-GNR-DC and 4T1-HANG-GNR-DC.

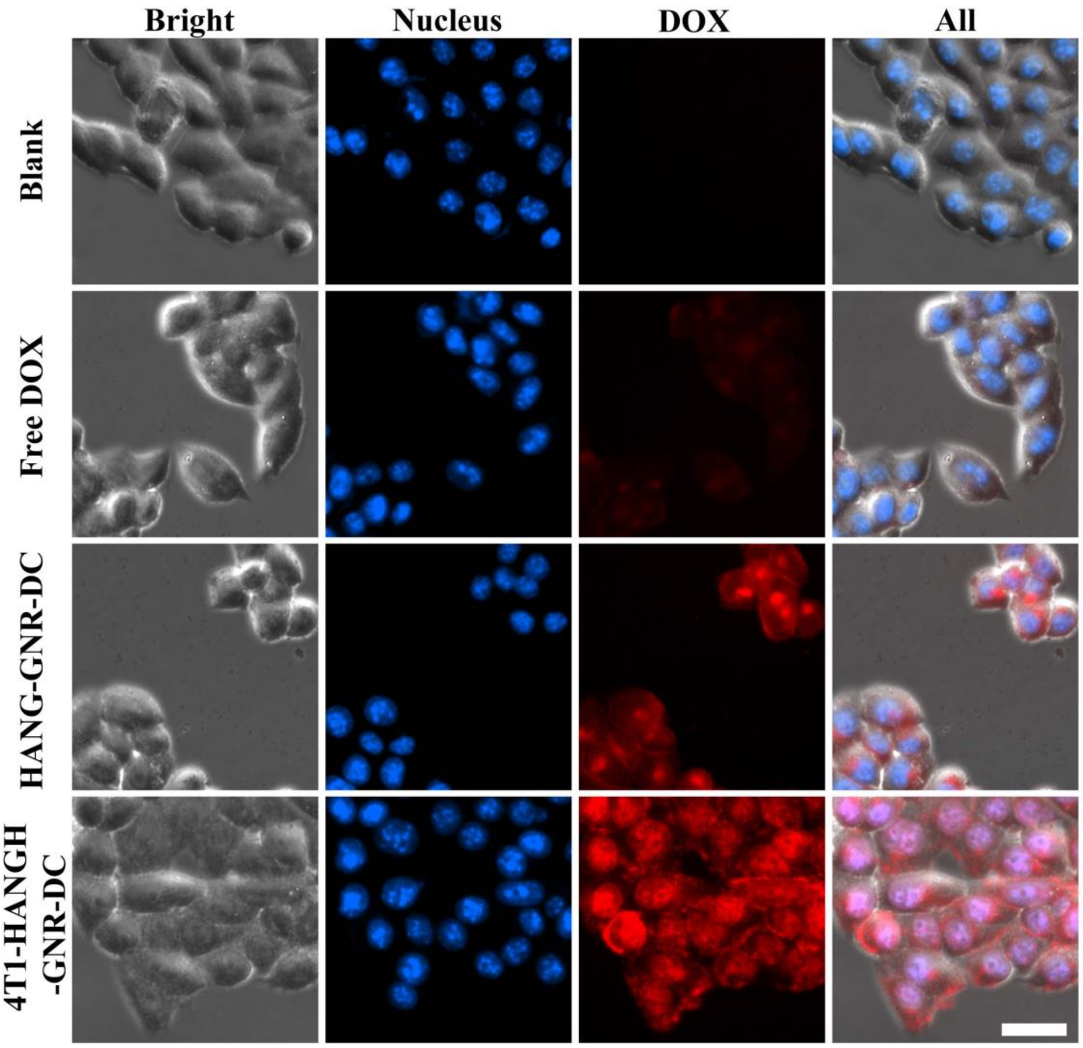

Figure S3. Cellular uptake of the different formulations on 4T1 cells after 8 h incubation. The nucleus was labeled by Hoechst 33342 (blue). Scale bar: 20 μm.

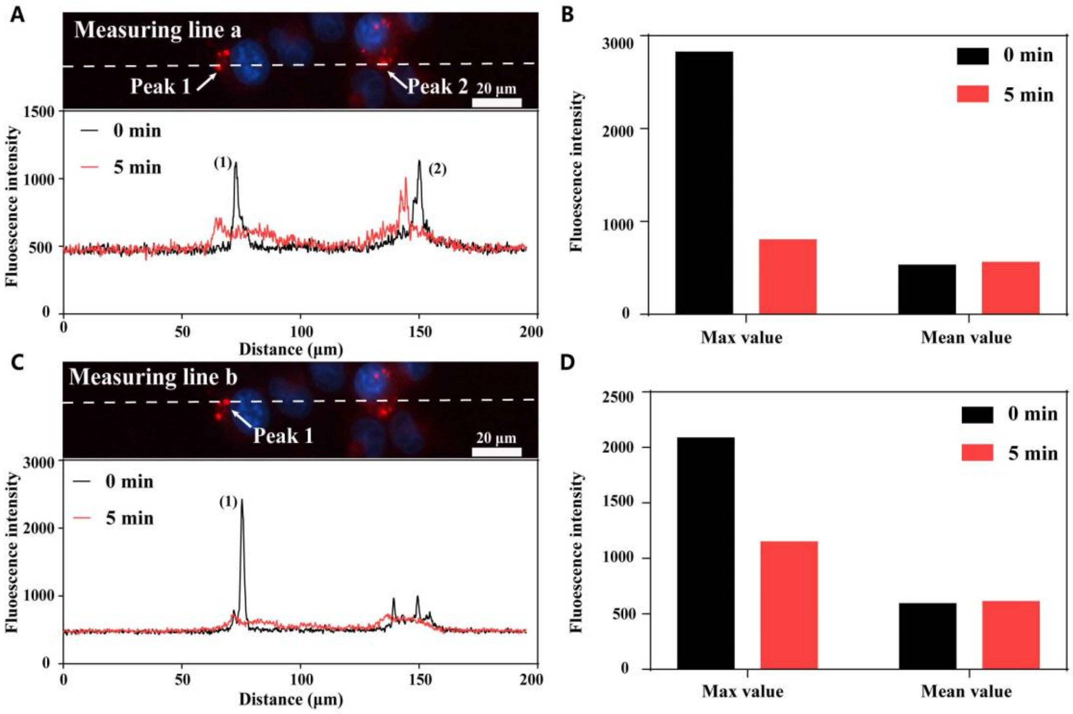

Figure S4. Fluorescence images of 4T1-HANG-GNR-DC internalized 4T1cells (A) before and (C) after 5 min of light irradiation. Corresponding max (mean) value of fluorescence intensity in region a (C) and region b (D).

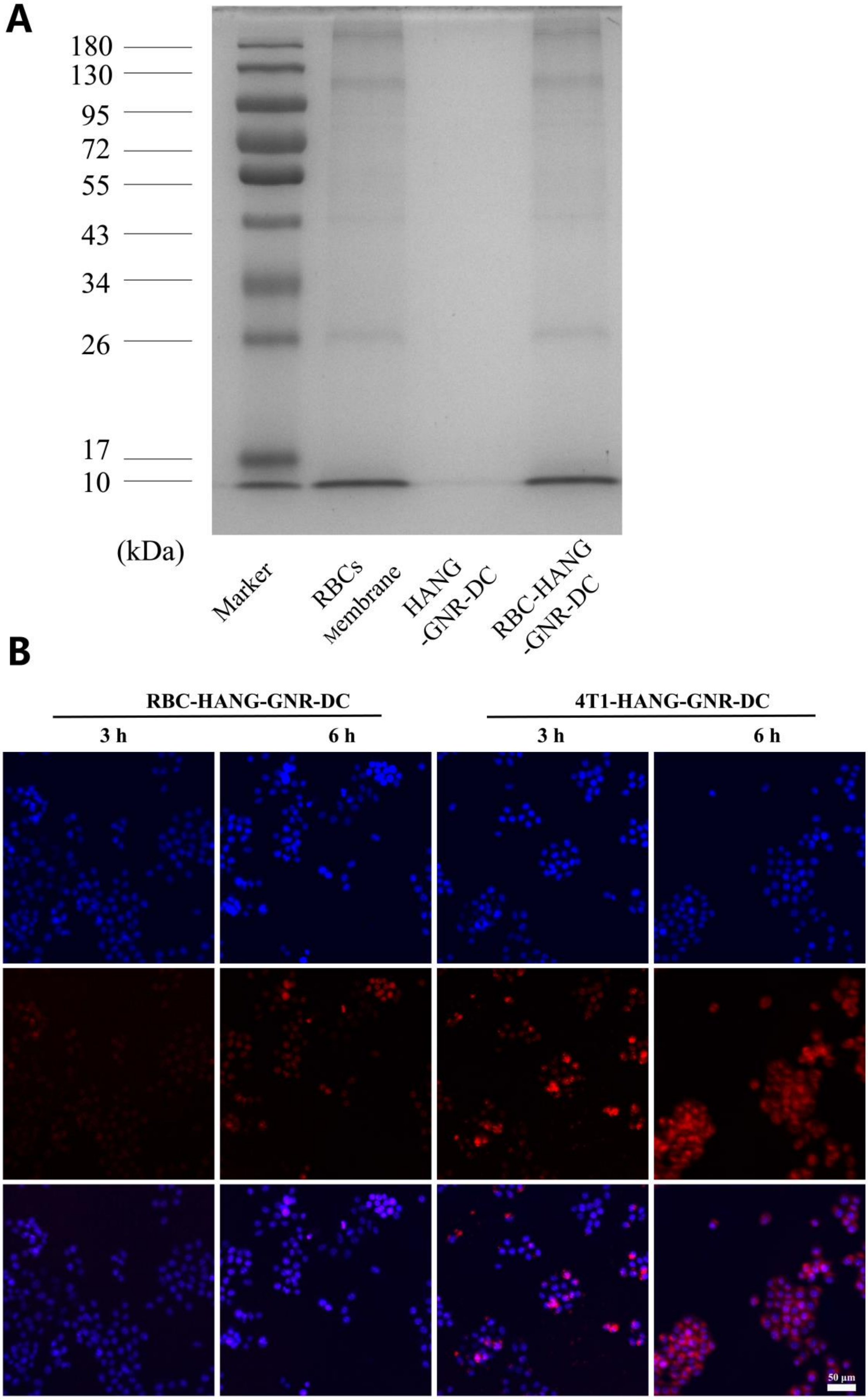
A
180
130
95
72
55
43
34
26
17
10
(kDa)
Marker
RBCs Membrane
HANG -GNR-DC
RBC-HANG -GNR-DC
B
RBC-HANG-GNR-DC
4T1-HANG-GNR-DC
3 h
6 h
3 h
6 h
Hoechst 33342
DOX
All
50 μm

Figure S5. A) SDS-PAGE analysis of marker, RBCs cell membranes, HANG-GNR-DC and RBC-HANG-GNR-DC. B) CLSM of 4T1 cells after incubation with RBC-HANG-GNR-DC and 4T1-HANG-GNR-DC for 3 or 6 h. Scale bar: 50.0 μm.

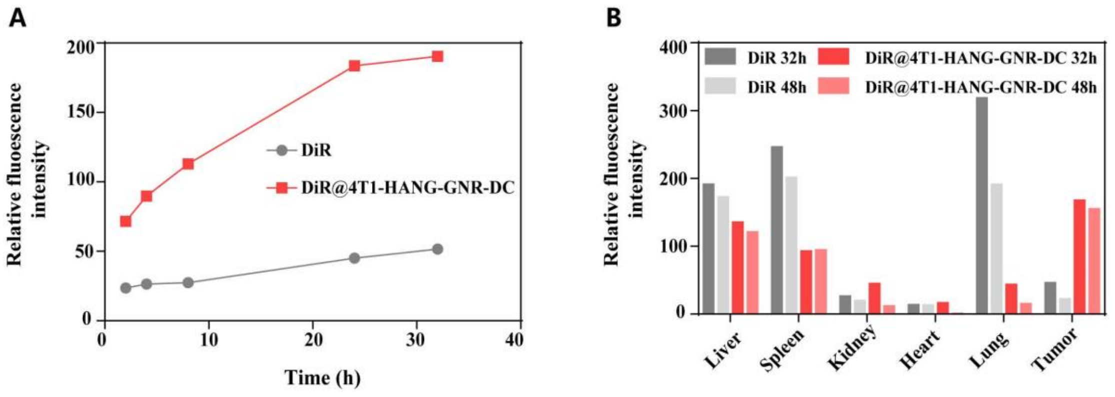

Figure S6. A) Semi-quantitative fluorescence of DiR@4T1-HANG-GNR-DC and DiR in the tumor at time points. B) Biodistribution of DiR@4T1-HANG-GNR-DC and DiR in tumor-bearing mice after intravenous injection (determined by fluorescence image ROI measurement of *ex vivo* organs).

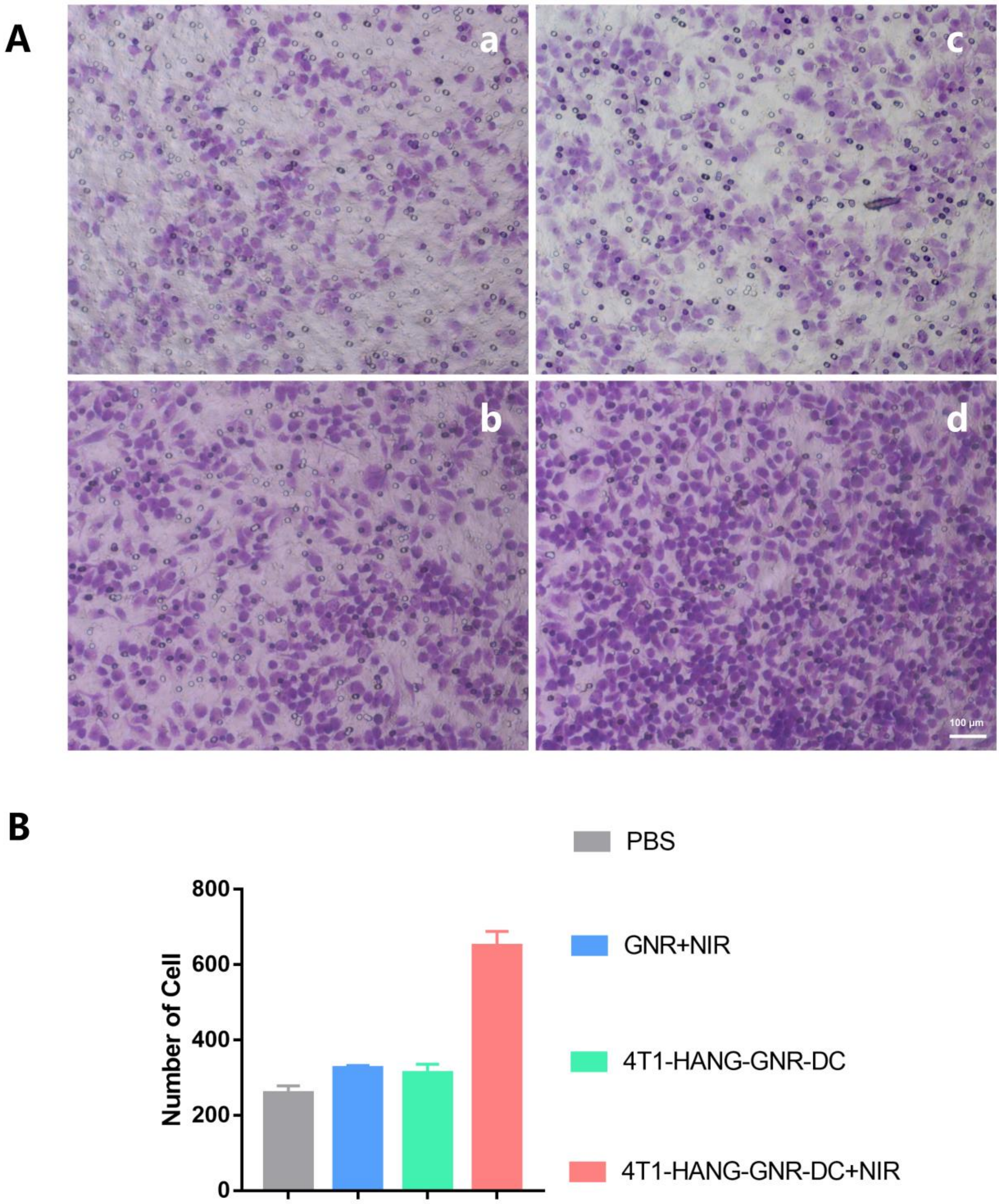

Figure S7. A) Migration of macrophages induced by hyperthermia therapy. Macrophages were stained with 0.1 % gentian violet. (a: PBS; b: GNR+NIR; c: 4T1-HANG-GNR-DC; d: 4T1-HANG-GNR-DC+NIR) Scale bar: 100.0 μm. B) Statistical analysis of the number of macrophages migrated below the culture chamber.

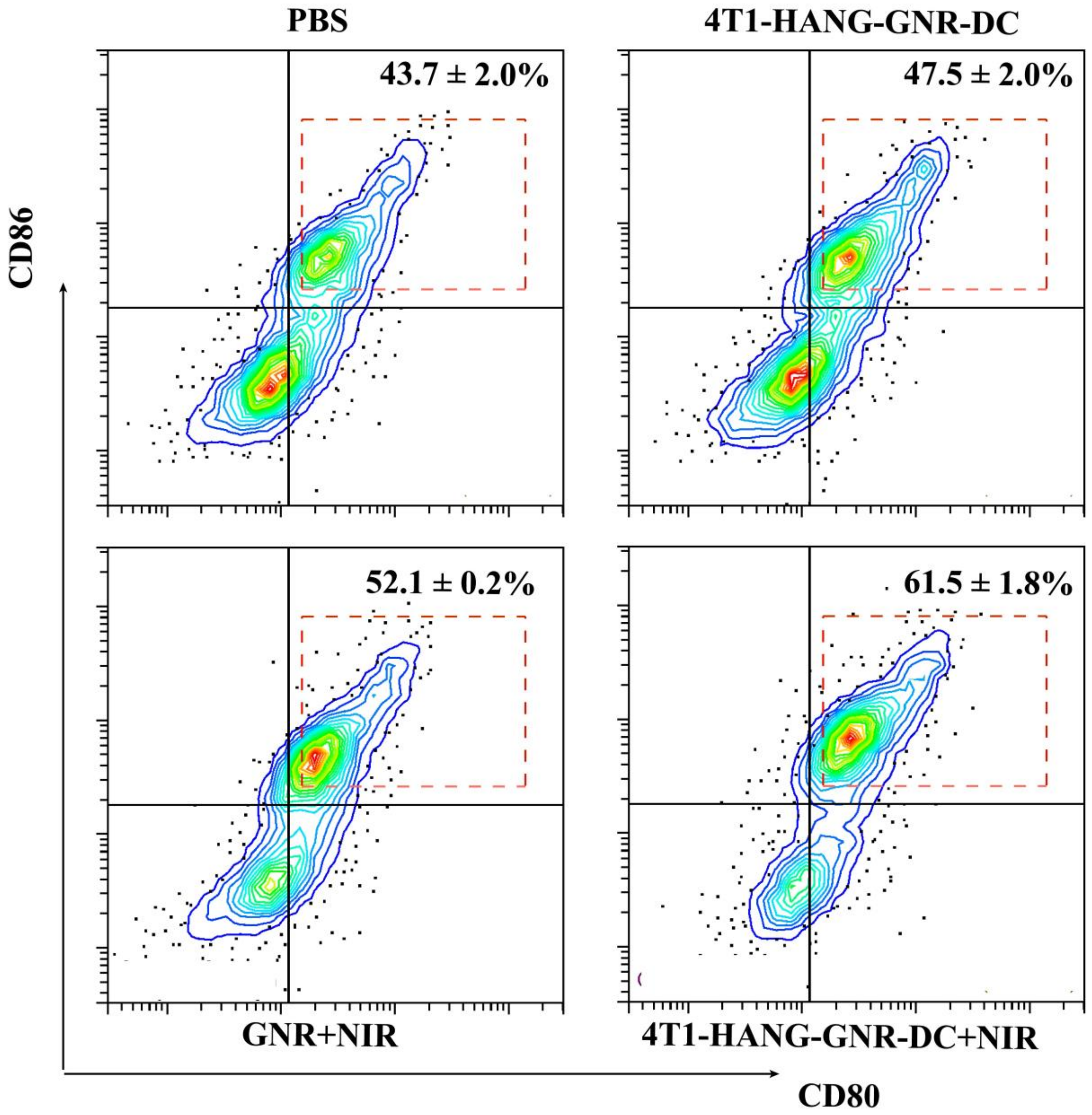

Figure S8. Flow cytometry analysis of anti-tumor hyperthermia threapy (4T1-HANG-GNR-DC) induced DC maturation (CD11c+CD80+CD86+).

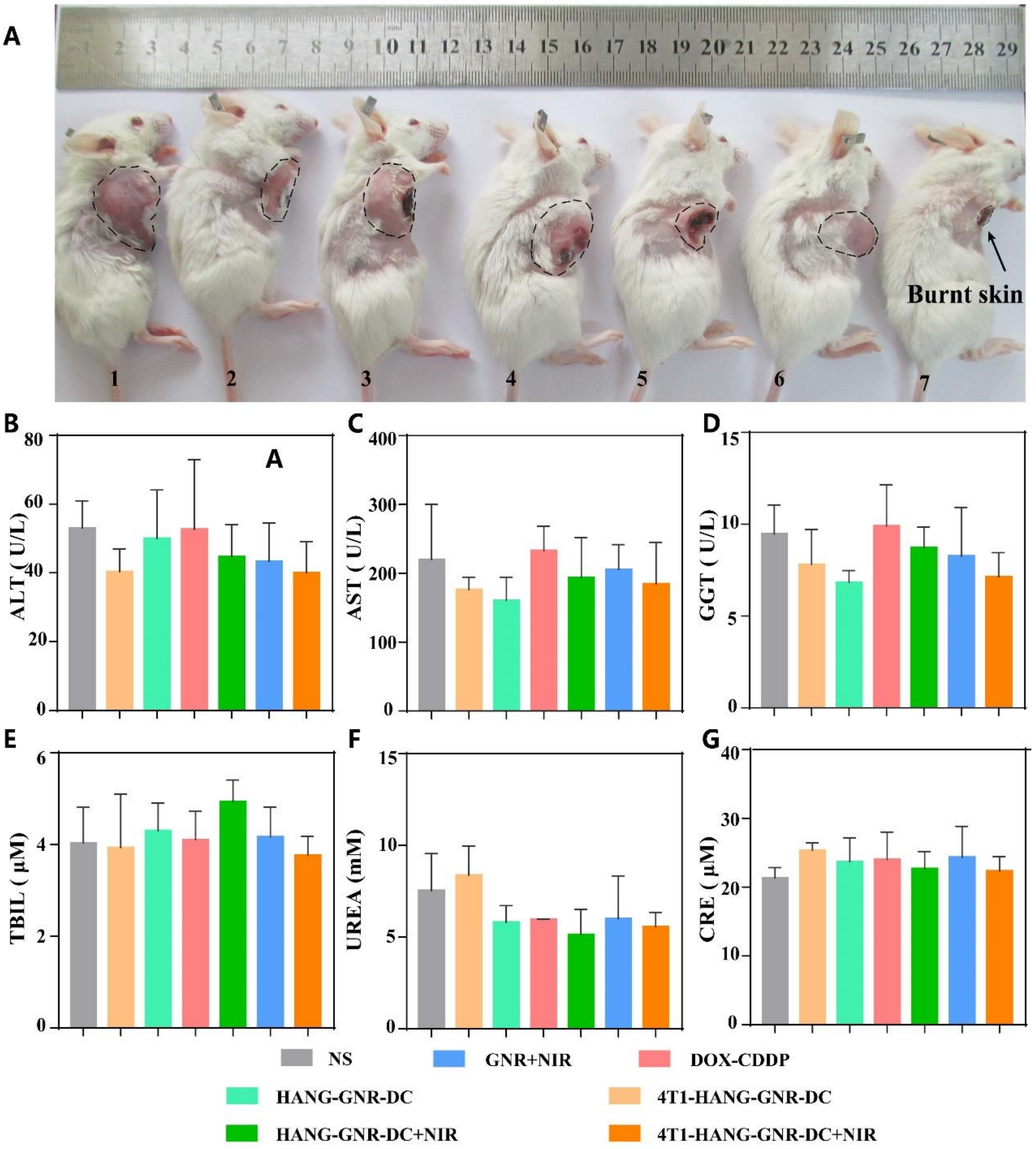

Figure S9. A) Photographs of 4T1 tumor-bearing mice after treatment (1: NS, 2: 4T1-HANG-GNR-DC, 3: HANG-GNR-DC, 4: DOX-CDDP, 5: HANG-GNR-DC+NIR, 6: HA-GNR+NIR, 7: 4T1-HANG-GNR-DC+NIR). Biochemical index including B) alanine aminotransferase (ALT), C) aspartate aminotransferase (AST), D) glutamyl transpeptidase (GGT), E) total bilirubin (TBIL), F) blood urea nitrogen (UREA), G) creatinine (CRE) of 4T1 tumor-bearing mice after treating for 16 days. (n=3)

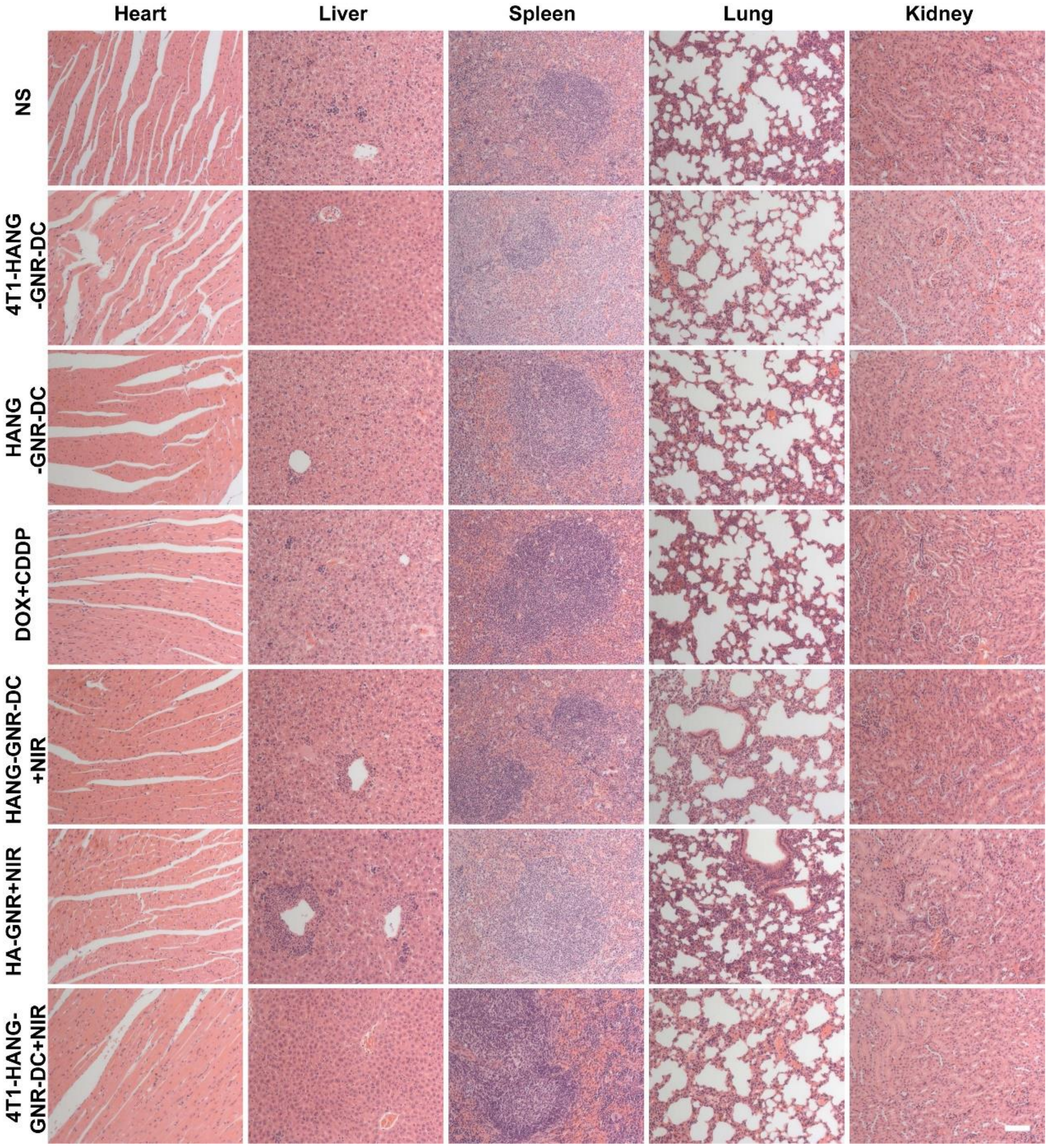

Figure S10. H&E staining of heart, liver, spleen, lung and kidney tissue slices from tumor-bearing mice after treatment for 16 days. Scale bar: 20 μm.
